# Notes on Preparations for the Sick

**Published:** 1913-06

**Authors:** 


### Notes on Preparations for the Sick.

**Phosphocose—Glycero-phosphate Somatose.**—The Bayer Co., London.—The various preparations of somatose have been noticed by us from time to time. The list now includes the guaiacol compound of calcium guaiacol-sulphonate called guycose, iron somatose, irocose=liquid iron somatose, liquid somatose, milk somatose; and to these is now added phosphocose. It is a brown syrupy liquid of pleasant flavour, and consists of a pleasantly-flavoured solution of somatose, containing sodium glycero-phosphate. It contains no alcohol or other preservative, but is sent out sterilised: it should therefore be prescribed and dispensed in the original bottles only. In cases of nervous debility and neurasthenia generally the combination of a soluble meat albumen with glycero-phosphate is an ideal fluid, which should have a very wide range of therapeutic utility.

**Horlick's Malted Milk. Lunch Tablets.**—Horlick's Malted Milk Co., Slough.—These tablets, five-eighths of an inch in diameter, are packed in a flat box, a quarter of an inch in thickness, so forming a vest pocket lunch for very busy people. They are supplied in natural flavour, or with chocolate, and the case is refilled from time to time from the larger glass jar. They may be swallowed or dissolved slowly in the mouth as desired.

---

**Luminal, Luminal Sodium, Afridol Soap.**—THE BAYER CO., London.—The luminal tablets contain a grain and a half of this powerful hypnotic, which is chemically phenyl-ethyl-malonyl-urea, a white odourless powder of somewhat bitter taste. It differs from veronal in having a phenyl group in place of the ethyl group.

The luminal sodium is a soluble form, which may be used hypodermically, the ordinary dose being 2.3 c.c. of 20 per cent. solution. The solution should be prepared in cool boiled water, and will keep for about eight days.

These drugs should be given cautiously, the maximum dose should not exceed twelve grains. In mental cases, alcoholics, drug habitués, etc., they have given excellent results, and may largely replace scopolamine.

Afridol soap is stated to be strongly germicidal, and thus useful for disinfecting the hands, instruments, and skin generally. It is not irritating, and does not injure instruments.

---

**Euthymol—Tooth Paste, Cold Cream, Shaving Cream, Talcum Powder, Tooth Powder, Bath Crystals.**—PARKE, DAVIS AND CO., London.—All these preparations have as their essential basis a proportion of this well-known antiseptic called euthymol. In the tooth paste it is equivalent in germicidal power to a 14 per cent. solution of carbolic acid, and although the mouth can never be absolutely sterilised, yet much can be done by chemical and mechanical methods combined to counteract the effects of pyorrhœa and other septic conditions of the mouth and throat. The euthymol paste is non-irritating and non-poisonous, but is pleasant to the taste, and thoroughly efficient as an oral germicide.

---

**Chrismol.**—ALLEN & HANBURY, London.—A liquid paraffin of the highest purity, intended for internal use. As such it may be of some mechanical advantage to the alimentary canal, and indirectly may improve nutrition; but we have always held that petroleum internally must be of little use, inasmuch as it passes through the alimentary canal unchanged. Patients say they derive benefit from its use, and that they gain in weight thereby: but much evidence is required to verify these statements. If paraffin be the thing, surely this preparation must be the best form of it.

---

**Novatophan.**—A. & M. Zimmermann, 3 Lloyd's Avenue, London, E.C.—In Dec., 1912, we noted the chief points relating to a phenylchinoline carbonic acid compound named atophan. It has been found to have a powerful influence on uric acid metabolism, increasing the uric acid elimination remarkably.

The newer form is the ethyl ester of methylated atophan, which is the equivalent in clinical effect, but without the bitter taste. It is not soluble, and therefore the tablet form is advised, six to ten being the usual daily dose (45 to 75 grains).

---

**Pond's Tampons.—Local Medication for Women.**—J. M. Richards, London.—These vaginal tampons are easily applied without speculum. Moistened in warm water for about ten seconds and then inserted, being held in place for a few moments, it will be found that the gelatine shell soon dissolves, the expanding wool forcing the medicated cone against the cervix, and dilating the vaginal walls. They are made in three sizes, and contain various medicated compounds such as ichthyol compound with boro-glyceride, protargol, tannin, and opium, belladonna, and hyoscyamin with a boro-glyceride base. The medicated tips are completely dissolved in about 24 hours, when the tampon should be changed.

---

**Ems Mineral Spring Salt Pastilles.**—Hertz & Co., 9 Mincing Lane, London.—This natural salt is obtained by evaporation of the water of the Ems spring at its source, under control of the Prussian State.

---

**Lactona.**—H. E. Matthews & Co., Clifton.—This is a tonic milk powder, prepared by a local firm, and differing from many similar preparations. In the first place our analysis showed it to contain over 20 per cent. of fat, and this alone places it in a class by itself. It is also extremely palatable, and is especially pleasant when milk is used as the vehicle for its administration. Unlike the majority of tonic foods the price is low, a half pound tin costing only 2s. The addition of 5 per cent. sodium glycerophosphate largely increases the nutritive value of the other constituents.

